# Colocalising proteins and polysaccharides in plants for cell wall and trafficking studies

**DOI:** 10.3389/fpls.2024.1440885

**Published:** 2024-09-11

**Authors:** Edwin R. Lampugnani, Staffan Persson, Allison M. L. van de Meene

**Affiliations:** ^1^ Menzies Institute for Medical Research, College of Health and Medicine, University of Tasmania, Hobart, TAS, Australia; ^2^ School of Health Sciences, University of Melbourne, Parkville, VIC, Australia; ^3^ Department of Medicine (RMH), Melbourne Medical School, University of Melbourne, Parkville, VIC, Australia; ^4^ AirHealth Pty Ltd., Parkville, VIC, Australia; ^5^ School of BioSciences, The University of Melbourne, Parkville, VIC, Australia; ^6^ Department of Plant and Environmental Sciences (PLEN), University of Copenhagen, Frederiksberg, Denmark; ^7^ Advanced Microscopy Facility (BioSciences Microscopy Unit and the Ian Holmes Imaging Centre, Bio21), The University of Melbourne, Parkville, VIC, Australia

**Keywords:** plant cell walls (PCWs), APEX2, antibodies, cellulose synthase, pectins, polysaccharides, electron microscopy, non-cellulosic polysaccharides (NCPs)

## Abstract

Plant cell walls (PCWs) are intricate structures with complex polysaccharides delivered by distinct trafficking routes. Unravelling the intricate trafficking pathways of polysaccharides and proteins involved in PCW biosynthesis is a crucial first step towards understanding the complexities of plant growth and development. This study investigated the feasibility of employing a multi-modal approach that combines transmission electron microscopy (TEM) with molecular-genetic tagging and antibody labelling techniques to differentiate these pathways at the nanoscale. The genetically encoded electron microscopy (EM) tag APEX2 was fused to *Arabidopsis thaliana* cellulose synthase 6 (*At*CESA6) and *Nicotiana alata* ARABINAN DEFICIENT LIKE 1 (*Na*ARADL1), and these were transiently expressed in *Nicotiana benthamiana* leaves. APEX2 localization was then combined with immunolabeling using pectin-specific antibodies (JIM5 and JIM7). Our results demonstrate distinct trafficking patterns for *At*CESA6 and *Na*ARADL, with *At*CESA6 localized primarily to the plasma membrane and vesicles, while *Na*ARADL1 was found in the *trans*-Golgi network and cytoplasmic vesicles. Pectin epitopes were observed near the plasma membrane, in Golgi-associated vesicles, and in secretory vesicle clusters (SVCs) with both APEX2 constructs. Notably, JIM7 labelling was found in vesicles adjacent to APEX2-*At*CESA6 vesicles, suggesting potential co-trafficking. This integrative approach offers a powerful tool for elucidating the dynamic interactions between PCW components at the nanoscale level. The methodology presented here facilitates the precise mapping of protein and polysaccharide trafficking pathways, advancing our understanding of PCW biosynthesis and providing avenues for future research aimed at engineering plant cell walls for various applications.

## Introduction

Biological organisms are a complex mix of proteins, nucleic acids, lipids and polysaccharides, each playing crucial roles in cellular functions. Fluorescent microscopy techniques offer a wealth of options for studying proteins in cell biology, including fluorescent-tagged proteins and antibody labelling. The visualization of polysaccharide biosynthesis, particularly in plant cells, presents unique challenges due to the paucity of molecular labelling options for polysaccharides as well as submicron vesicles that often fall below optical resolution limits, the thick cell walls of these vesicles and the light scattering properties of their vacuoles. To address this, we investigated the use of a molecular-genetic tag for electron microscopy that was co-labelled with polysaccharide antibodies to reveal the trafficking of proteins and polysaccharides in plants at the nanoscale.

Plant cell walls (PCWs) are indispensable for both plant life and human society and serve diverse functions ranging from structural support to various industrial applications. Beyond their pivotal roles in plant biology, cell walls have found widespread utilisation in a wide variety of areas including as dietary fibre for human nutrition, as feed for animals, paper production, textiles in clothing and as sustainable building materials.

The complexity of plant cell walls stems from their intricate composition, which includes a rich assortment of polysaccharides, proteins, and other macromolecules organized into primary and secondary wall layers. The primary wall, positioned outside the plasma membrane, forms the initial interface between the cell and its environment. The primary wall is composed of cellulose microfibrils, typically the most abundant polymer, which are laid down first as the cells are growing. The secondary cell walls are strengthened by phenolic polymers such as lignin (Doblin et al., 2010; Lampugnani et al., 2018).

In the primary PCW, cellulose, a polymer of (1→4)-β-D-glucose units, is intertwined with an array of non-cellulosic polysaccharides (NCPs), such as xyloglucans, xylans and pectins, each imparting specific properties to the cell wall matrix. Xylans, with their β-(1→4)-linked xylose residues and diverse side-chain decorations, contribute to the structural integrity and hydration properties of the cell wall (Curry et al., 2023). Meanwhile, pectins, rich in galacturonic acid, regulate cell wall porosity and play pivotal roles in cell adhesion, signalling, and developmental processes (Lee et al., 2013; Verhertbruggen et al., 2013; Mariette et al., 2021). The interplay between these components forms the basis of cell wall architecture and influences cell shape, growth, and response to environmental cues, thereby impacting plant form and structure.

Plant cell wall polysaccharides are synthesized by a diverse array of glycosyltransferase (GT) enzymes that catalyse the formation of glycosidic bonds between sugar donors, leading to the network of polysaccharides found in cell walls (Lairson et al., 2008). The complexity of cell wall composition arises from the dynamic interactions among various GTs, generating a wide range of linkages crucial for cell wall integrity and function. For instance, NCPs undergo processing within the Golgi Apparatus (GA) where diverse polysaccharides are synthesised and modified by specific GT enzymes (Pauly et al., 2013).

Cellulose is synthesised by cellulose synthase A enzymes (CESAs), which assemble into ring-shaped structures known as cellulose synthase complex (CSCs). These CSCs traverse the plasma membrane and are guided by microtubules in the cytoplasm while they simultaneously extrude the cellulose microfilament by adding new glucose molecules to the growing polysaccharide chain (Gonneau et al., 2014; McFarlane et al., 2014; Nixon et al., 2016; Turner and Kumar, 2018; Pedersen et al., 2023). The formation and activity of CSCs are tightly regulated processes essential for cell wall biosynthesis and architecture.

Following synthesis, CESAs undergo processing within the GA and transit through the *trans*-Golgi network (TGN) before being delivered to the plasma membrane. This trafficking process involves specialised compartments known as small CESA compartments (SmaCCs) (Gutierrez et al., 2009) or microtubule-associated cellulose synthase compartments (MASCs) (Crowell et al., 2009), which facilitate the transport of CESAs to their destination via secretory vesicles. The precise coordination of CESA trafficking is vital for the spatial and temporal regulation of cellulose deposition, ensuring proper cell wall formation and functionality. Live cell, fluorescence microscopy has enabled the visualization and characterization of these dynamic trafficking pathways, providing valuable insights into the mechanisms underlying cell wall biosynthesis.

In this paper, we focus on cellulose as well as pectin, a complex and structurally diverse NCP composed of various components including, but not limited to, three main galacturonic acid-rich polysaccharides: homogalacturonan (HG), rhamnogalacturonan I (RGI) and rhamnogalacturonan II (RGII) (Ridley et al., 2001; Willats et al., 2001; Mohnen, 2008). L-arabinose is a saccharide found in multiple linkages in side-branches of the RGI polymer. Arabinose residues can also be found in “free” arabinans, contributing to the structural diversity of pectic polysaccharides. The significance of L-arabinose extends beyond its structural role, as highlighted by recent research elucidating its importance in plant development and physiology (Mariette et al., 2021). Arabinose-containing polymers, such as arabinans, play critical roles in cell wall dynamics, influencing growth, structure, and adaptive responses in plants. These polymers are synthesized and modified by a network of enzymes, including ARABINAN DEFICIENT 1 (ARAD1), a member of the GT47 family. ARAD1 functions as an arabinan α-1,5-arabinosyltransferase and contributes to the biosynthesis of arabinan side chains in *Arabidopsis* RG1 (Harholt et al., 2006) and “free” arabinan in pollen tubes of *Nicotiana alata* (Lampugnani et al., 2016). It was found to be a Type-II membrane protein localised to Golgi vesicles (Harholt et al., 2006) and lumen (Lampugnani et al., 2016) with arabinan-containing pectic polysaccharide components delivered to the cell wall presumably via secretory vesicles. In *Nicotiana alata*, the presumed arabinosyltranserase is called *Na*ARADL1 and in this study, it was used as the representative GT in the pectic polysaccharide pathway.

Although both the CESAs and the *Na*ARADL1 proteins have been localised within the GA, it remains unclear whether the trafficking routes of the CSCs, SmaCCs and MASCs identified using fluorescence microscopy align with the secretory vesicle pathway observed for pectic polysaccharides *en route* to the plasma membrane and cell wall. Here we aim to bridge this knowledge gap by proposing a methodology to differentiate between the trafficking pathways of proteins and polysaccharides through the leveraging of transmission electron microscopy (TEM) methods.

TEM methods have been instrumental in tagging various NCPs using cell wall antibodies, allowing visualization within key intracellular compartments such as the GA, *trans*-Golgi network (TGN), secretory vesicles, and the cell wall itself (Barany et al., 2010; Pattathil et al., 2010; Wilson et al., 2015; van de Meene et al., 2017; Rydahl et al., 2018; van de Meene et al., 2021). While freeze fracture TEM has successfully identified CSCs in vesicles of the desmid *Micrasterias denticulata* (Giddings et al., 1980) and in the plasma membrane of the *Physcomitrella patens* moss protonema (Nixon et al., 2016), their detection using standard TEM cytological methods involving fixation, embedding, and sectioning has proven challenging.

To address this limitation, a genetically encoded EM tag, APEX2 (Lam et al., 2015), has been used. APEX2 is an engineered ascorbate peroxidase derived from the soybean APEX protein (Rhee et al., 2013; Hung et al., 2014) that catalyses the generation of short-lived, highly reactive, and membrane-impermeant radicals in living cells (Lam et al., 2015). The tag is suitable as both an electron microscopy tag, where it forms a precipitate in the cell after diaminobenzidine-osmium tetroxide oxidation, and for proteomics studies. This approach has been successfully used to identify interactors in the human mitochondrial matrix and intermembrane space (Rhee et al., 2013; Hung et al., 2014; Lam et al., 2015).

To elucidate whether CESA enzymes follow the same or distinct secretory routes compared to enzymes associated with the synthesis of pectic NCPs, we genetically tagged CESA6 and *Na*ARADL1 with the APEX2 tag and then transiently expressed these constructs separately in *Nicotiana benthamiana* leaves. The excised leaf samples were subsequently processed for APEX2 oxidation and TEM. After sectioning, the samples were antibody labelled using the pectin epitopes JIM5 and JIM7 (Knox et al., 1990). The results obtained show that CESA6 is trafficked separately to the pectic polysaccharides identified by these antibodies. The *Na*ARADL1-APEX2 density localised to the GA and was found in some secretory vesicles. These findings indicate differential trafficking pathways for CESAs and pectic NCPs, highlighting the complexity and specificity of intracellular transport mechanisms and shedding light on the dynamic interplay driving cell wall synthesis and remodelling processes.

## Materials and methods

### Plant growth & transient expression

*N. benthamiana* plants were grown in soil in a growth cabinet with continuous cool-white fluorescent lights at 20-26°C with a 16:8 hour light:dark cycle.

### Construct development and transient expression

The APEX2 tag sequence (**Supplementary 1**) was synthesized with alanine linkers on the N and C terminus to facilitate cloning of fusions with genes of interest. The gene block was cloned into the *Kpn*I and *Bam*HI sites of the binary vector pFUERTE, which contains the CaMV35S promoter and the 3′ OCS terminator sequence (Lampugnani et al., 2016), using New England Biolabs HiFi DNA assembly reagents and the protocol specified by the manufacturer. This generated the construct 35S:pcoAPEX2. To generate translational fusions for this project, the *Arabidopsis* CESA6 (*At*CESA6; AT5G64740) and *Na*ARADL1 were each amplified using the primers outlined in **Supplementary** **Table S1** **.2**. The resulting PCR products and digested vectors were cleaned using a Qiagen PCR clean-up kit and combined using New England Biolabs HiFi DNA assembly reagents following the protocol specified by the manufacturer to generate the following constructs: 35S:pcoAPEX2(empty), 35S:pcoAPEX2-*At*CESA6 (APEX2-*At*CESA6) and 35S:*NaARADL1*-pcoAPEX2 (*Na*ARADL1-APEX2).

Transient expression in *N. benthamiana* leaves was carried out as described previously (Lampugnani et al., 2016). Transformations were carried out at least in triplicate and entire leaves were infiltrated. Three days after infiltration a small sample (~1 cm^2^) of each leaf was excised and processed for APEX2 oxidation and electron microscopy as described below. Each infiltration was performed on duplicate leaves and repeated three times.

### APEX2-DAB oxidation and electron microscopy

The APEX2 transformed leaf segments of the *N. benthamiana* lines were processed for electron microscopy as outlined in previous APEX2 studies (Martell et al., 2012; Hung et al., 2014; Lam et al., 2015) with slight modifications for the plant material. Briefly, the leaf segments were fixed with 2.5% glutaraldehyde (Electron Microscopy Sciences) in phosphate buffered saline (PBS) at 4°C overnight. After fixation, all processing was done on ice. The leaf tissue was rinsed 5 x 5 min in PBS followed by quenching of the glutaraldehyde with 20 mM glycine in PBS for 30 min. The samples were again rinsed 5 x 5 min with PBS after which the samples were placed in a solution of 1.4 mM 3,3’-diaminobenzidine (DAB) free base (Sigma) dissolved in HCl and 10 mM H_2_O_2_ in cold PBS for 15 min for the APEX2-catalysed polymerisation of DAB. The samples were then rinsed 3 x 10 mins with cold PBS and 2 x 10 mins in double distilled H_2_O (ddH_2_O). The leaf tissue was post-fixed with 1% OsO_4_ for 30 mins, which stained the DAB polymers, and was then rinsed again 5 x 10 mins in ddH_2_O before placing into 1% aqueous uranyl acetate overnight. The following day, the leaf tissue was rinsed 5 x 10 mins in ddH_2_O and dehydrated in an ethanol series of 10%, 20%, 40%, 60%, 80%, and 3 x 100% for 1 hour at each dilution except for the last change of 100% ethanol, which was left overnight. The samples were then infiltrated with LR White resin (ProScitech) at 25%, 50%, 75% and 3 x 100% steps for 8-12 hours at each percentage step. The samples were then polymerised at 52°C for 24 hours before being cut into 70 nm sections with a Leica Ultracut 7 ultramicrotome (Leica Microsystems) and collected on Formvar coated gold grids. Sections were either post-stained with 1% uranyl acetate and lead citrate, or antibody labelled first and then post-stained.

### Immunogold labelling

Sections of each line were immunolabelled with cell wall antibodies to label for pectin epitopes including JIM5 and JIM7. All antibodies were from Plant Probes (University of Leeds). The carbohydrate binding module 3a (CBM3a), which has traditionally been used to bind to crystalline cellulose (Blake et al., 2006), was not used in this study as CBM3a has also been found to bind to xyloglucan (Hernandez-Gomez et al., 2015), which may lead to labelling in the GA.

Briefly, the sections were incubated in the blocking buffer of 1% bovine serum albumin (BSA) in PBS for 30 mins after which the sections were incubated overnight at 4°C in the primary antibody at a dilution of 1:20. The following day, the sections were washed 3 x 2 mins in PBS and 2 x 2 mins in ddH_2_O before being incubated with the anti-rat 18 nm gold secondary antibody (Jackson ImmunoLabs) with a dilution of 1:50. The negative control omitted the primary antibody.

### Imaging and image analysis

The sections were imaged on an FEI Tecnai Spirit TEM (Thermofisher Scientific) equipped with an FEI Eagle CCD camera. For each transformed line, three biological replicates were imaged for each immunogold experiment (i.e., negative control, JIM5 and JIM7 labelling). Images were randomly acquired of cytoplasm, cell wall and the surrounding formvar. At least 10 GA were imaged for each biological replicate, but not all GA showed gold labelling. The percentages given indicate the abundance of the gold particles in each cellular compartment measured. Images were analysed using the image analysis program FIJI (Schindelin et al., 2012).

## Results

### APEX2 constructs

**Figure 1:**
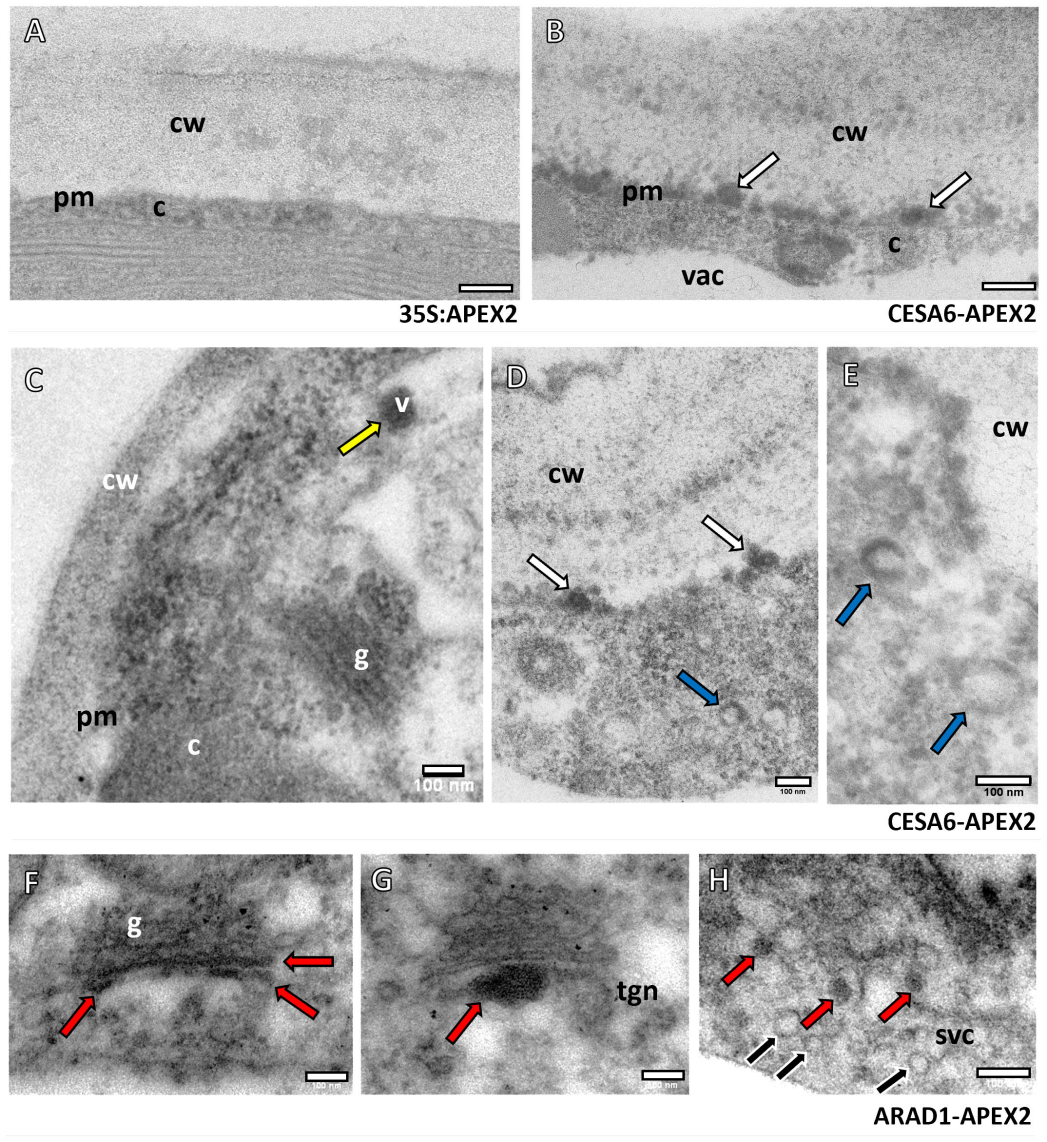
Subcellular localisation of APEX2-*At*CESA6 and *Na*ARADL1-APEX2 constructs using TEM. **(A)** The control construct 35S:APEX2 with no electron dense labelling adjacent to the cell wall (cw). **(B)** The APEX2-*At*CESA6 sample with electron dense labelling at a comparative region (white arrows). **(C–E)** Further labelling of the APEX2-*At*CESA6 construct in vesicles (v), adjacent to the cell wall (white arrows), and vesicles with what appears to be a translucent centre with electron dense labelling around the outside (blue arrows). **(F–H)** The *Na*ARADL1-APEX2 labelling showed electron dense labelling in the *trans*-Golgi, the TGN and in vesicles in the cytoplasm (red arrows). Multiple, non-labelled vesicles (black arrows) were observed in the same region as *Na*ARADL1-APEX2 positive vesicles in a possible SVC. cw, Cell wall; c, cytoplasm; vac, vacuole; v, vesicle; g, Golgi Apparatus; tgn, *trans*-Golgi network; svc, secretory vesicle cluster. Scale bars = 100 nm.

The APEX2-*At*CESA6 and *Na*ARADL1-APEX2 constructs were transiently transformed into *N. benthamiana*, and samples were subsequently processed for oxidation using DAB. DAB oxidation resulted in the formation of a precipitate, enhancing contrast for electron microscopy analysis. Our observations revealed distinct subcellular localization patterns: APEX2-*At*CESA6 were predominantly localised to the plasma membrane and vesicles in the cytoplasm, while *Na*ARADL1-APEX2 was primarily localised to the GA, the *trans*-Golgi network (TGN) and vesicles (**Figure 1**). In contrast, the control construct 35S:APEX2 (**Figure 1A**) did not exhibit the electron dense labelling observed in the experimental samples (**Figures 1B–H**). This contrast was particularly evident when comparing regions adjacent to the cell wall, where APEX2-*At*CESA6 showed intense electron-dense labelling (**Figure 1B**). Moreover, APEX2-*At*CESA6 expression was detected within vesicles that appeared to be entirely electron dense (**Figures 1C, D**) and also in the outer membrane region of vesicles where the centre was electron translucent (**Figures 1D, E**). No discernible labelling of APEX2-*At*CESA6 was observed in the GA in this study.

In comparison, the *Na*ARADL1-APEX2 construct (**Figures 1F–H**) exhibited distinct localisation patterns, with labelling detected within the *trans*-Golgi cisternae (**Figure 1F**), the TGN (**Figure 1G**) and in cytoplasmic vesicles (**Figure 1H**). Notably, our observations revealed heterogeneity in vesicular populations, with some vesicles displaying electron density indicative of protein labelling, while others lacked such density, suggesting differential labelling within distinct vesicular populations.

### Antibody labelling combined with APEX2 localisation

To assess whether the APEX2 constructs co-localised with NCPs as anticipated, we employed the pectin-associated antibodies JIM5 (recognising low degree of esterified homogalacturonan) (Knox et al., 1990; Willats et al., 2000) and JIM7 (detecting esterified homogalacturonan) (Knox et al., 1990; Willats et al., 2000). While LM19 (Verhertbruggen et al., 2009) has emerged as the preferred antibody for detecting unesterified pectins (Knox, 2021), the results obtained using the JIM5 antibody were consistent with those of LM19, hence JIM5 was employed in this study. Cell wall labelling functioned as the internal positive control, while omission of the primary antibody functioned as the negative control.

**Figure 2:**
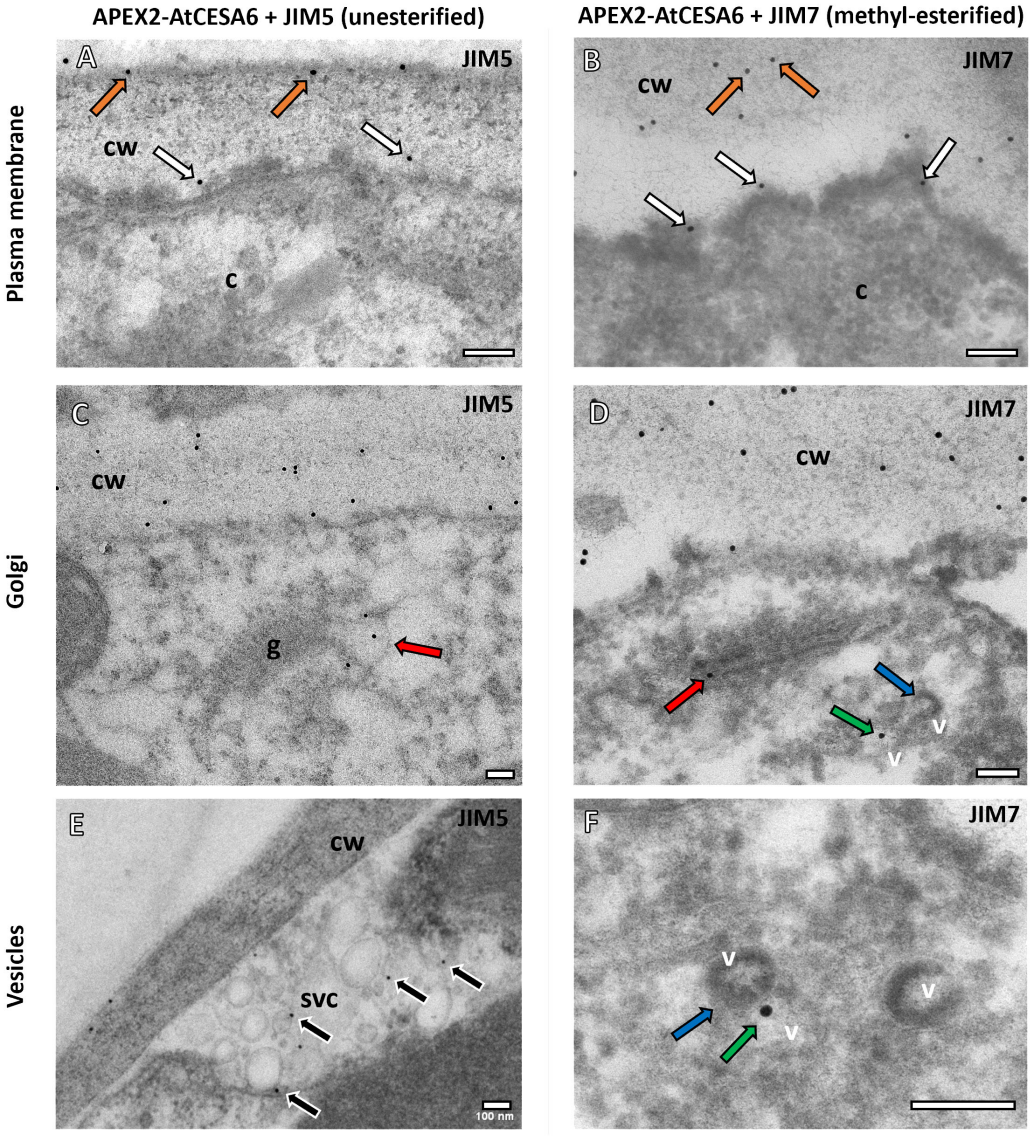
Subcellular localisation of APEX2-*At*CESA6 and pectin antibodies using TEM. **(A, B)** Both the JIM5 **(A)** and JIM7 **(B)** antibodies immunogold labelled the cell wall (orange arrows) and the APEX2 densities adjacent to the plasma membrane (this immunogold shown with white arrows) in the APEX2-*At*CESA6 line. **(C, D)** The GA were found to have both JIM5 **(C)** and JIM7 **(D)** antibody labelling in the *trans*-Golgi cisternae (red arrows). **(E, F)** Immunogold labelling was also observed in secretory vesicles clusters **(E)** and in vesicles (green arrows) adjacent to APEX2-positive vesicles (blue arrows) **(D, F)**. cw, Cell wall; c, cytoplasm; v, vesicle; g, Golgi Apparatus. Scale bars = 100 nm.

The colocalization analysis revealed distinct patterns of association between the JIM5 antibody and APEX2-*At*CESA6 (**Figures 2A, C, E**). Specifically, the JIM5 antibody labelling was observed in close proximity to the plasma membrane and closely juxtaposed with the APEX2-*At*CESA6 densities (**Figure 2A**). Additionally, JIM5 labelling was detected in vesicles on the *trans*-Golgi cisternae (**Figure 2C**) as well as vesicles found in secretory vesicle clusters (SVCs) (**Figure 2E**). These results were obtained from counting 1420 gold particles, 81% of which were located on the cell wall, 5% in proximity to the APEX2-*At*CESA6 plasma membrane densities, 1% in the TGN, 2.5% in the APEX2-CESA6 positive vesicles, 1% in the non-APEX2 positive vesicles and 2.4% in the SVCs. The remainder of the gold particles were found in the cytoplasm (3%) or as background on the formvar or vacuole. While these non-specific percentages may seem high, there was far more area imaged of the cytoplasm, vacuoles and the background Formvar than the areas of the GA and vesicles suggesting a higher overall density on the GA and vesicles than background labelling.

Conversely, the JIM7 antibody labelling (**Figures 2B, D, F**) exhibited a slightly greater overlap with the APEX2-*At*CESA6 densities at the plasma membrane compared to the JIM5 antibody (**Figure 2B**). Similar to JIM5, JIM7 labelling was observed within vesicles associated with the *trans*-Golgi cisternal vesicles (**Figure 2D**). Interestingly, the JIM7 immunogold labelling was also located in electron-lucent vesicles adjacent to vesicles containing APEX2-*At*CESA6 electron density (**Figures 2D, F**). These results were obtained from counting 794 gold particles. Again 80% of the gold labelling was quantified on the cell wall, but 9% was observed in proximity to the APEX2-*At*CESA6 densities at the plasma membrane, 1% on the GA, 1% on the APEX2-*At*CESA6 positive vesicles, 3% on non-APEX2 postive vesicles and 1.5% in SVCs. The remainder of the gold particles were observed in the cytoplasm, vacuoles or on the background formvar.

**Figure 3:**
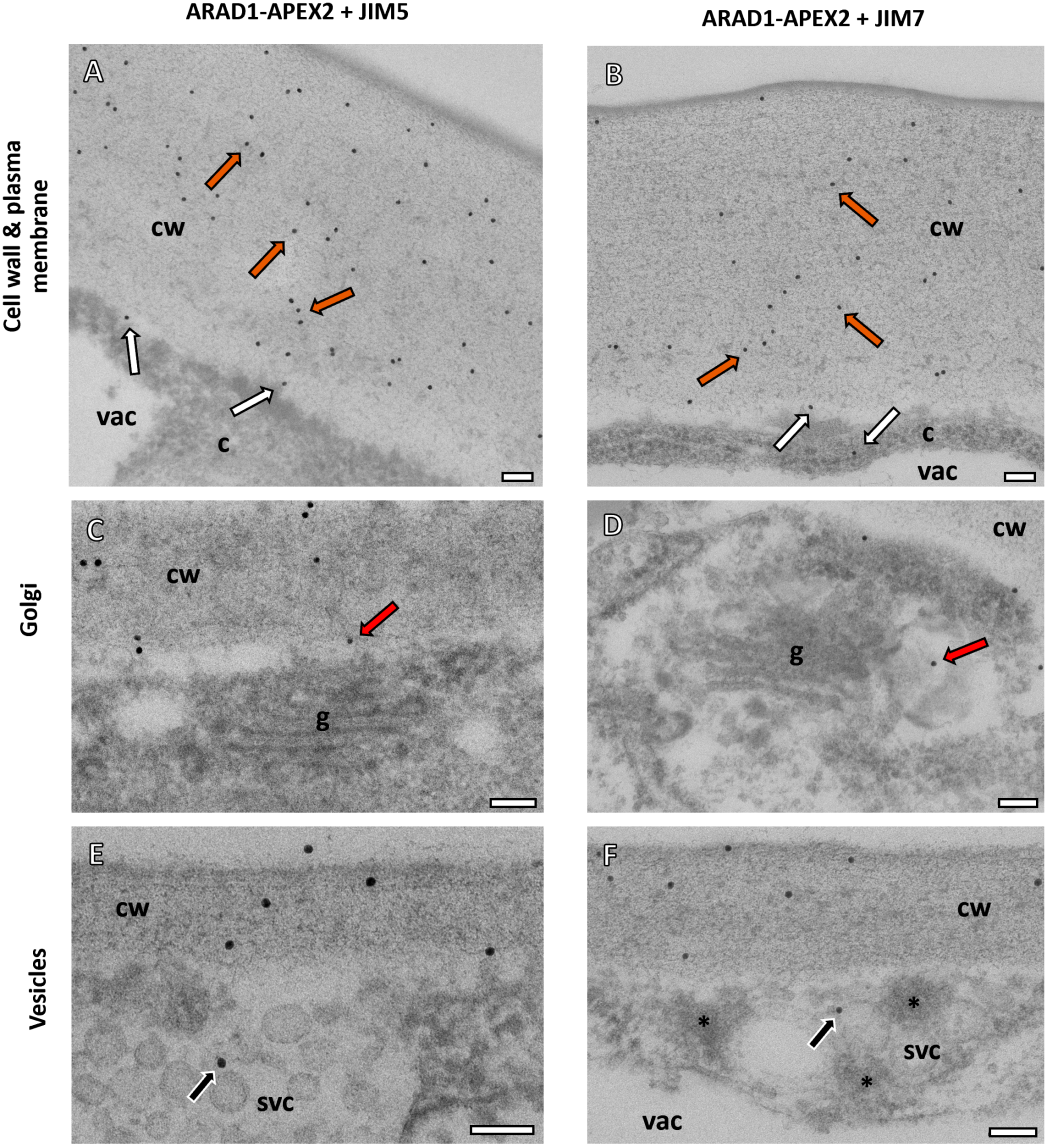
Subcellular localisation of *Na*ARADL1-APEX2 and pectin antibodies using TEM. **(A, B)** In the APEX2-*Na*ARADL1, both the JIM5 **(A)** and JIM7 **(B)** antibodies labelled the cell wall (orange arrows). Some immunogold labelling was observed near the plasma membrane and in the cytoplasm (white arrows). **(C, D)** The GA were closely associated with labelling for both JIM5 **(C)** and JIM7 **(D)** antibodies in vesicles associated with the *trans*-Golgi cisternae (red arrows). **(E, F)** Immunogold labelling for both JIM5 and JIM7 was also observed in secretory vesicles clusters **(E, F)**. Possible NaARADL1-APEX2 dense vesicles (asterisks) were found in the SVCs **(F)**. cw, Cell wall; c, cytoplasm; vac, vacuole; g, Golgi Apparatus. Scale bars = 100 nm.

In the *Na*ARADL1-APEX2 samples, both the JIM5 and JIM7 antibodies consistently labelled the cell walls (**Figures 3A, B**). Additionally, labelling was observed near the plasma membrane and within the cytoplasm (**Figures 3A–D**) as well as in Golgi associated vesicles (**Figures 3C, D**) and secretory vesicle clusters (**Figures 3E, F**) where *Na*ARADL1-APEX2 densities were also observed. These results were obtained from 519 JIM5- and 463 JIM7-associated immunogold labels respectively. For the JIM5 and JIM7 labelling, 81% of the gold particles were clearly on the cell wall and 9% were near the plasma membrane, 5% of the labels were found in vesicles or SVCs for JIM5 and 3% for JIM7 labelling. The remainder were in the cytoplasm, the vacuole or on the background Formvar grid.

## Discussion

The aim of this study was to explore a multi-pronged approach to investigate the trafficking pathways of PCW polysaccharides at the nanoscale in plants. By analysing the trafficking pathways of PCW synthases using the EM-tag APEX2 (Lam et al., 2015) in the plant transient transformation system of *N. benthamiana*, in conjunction with PCW antibodies, we sought to elucidate whether the trafficking pathway of non-cellulosic polysaccharides is different and independent of the cellulose trafficking pathway. Specifically, we focused on the localization of cellulose synthase (*At*CESA6) and arabinan transferase (*Na*ARADL1) in plant cells, combined with polysaccharide localisations using PCW antibodies to determine the associations of these components. Here, we discuss the implications of our findings and their significance in the context of PCW biosynthesis and trafficking, as well as the methodology employed and its potential applications.

**Figure 4:**
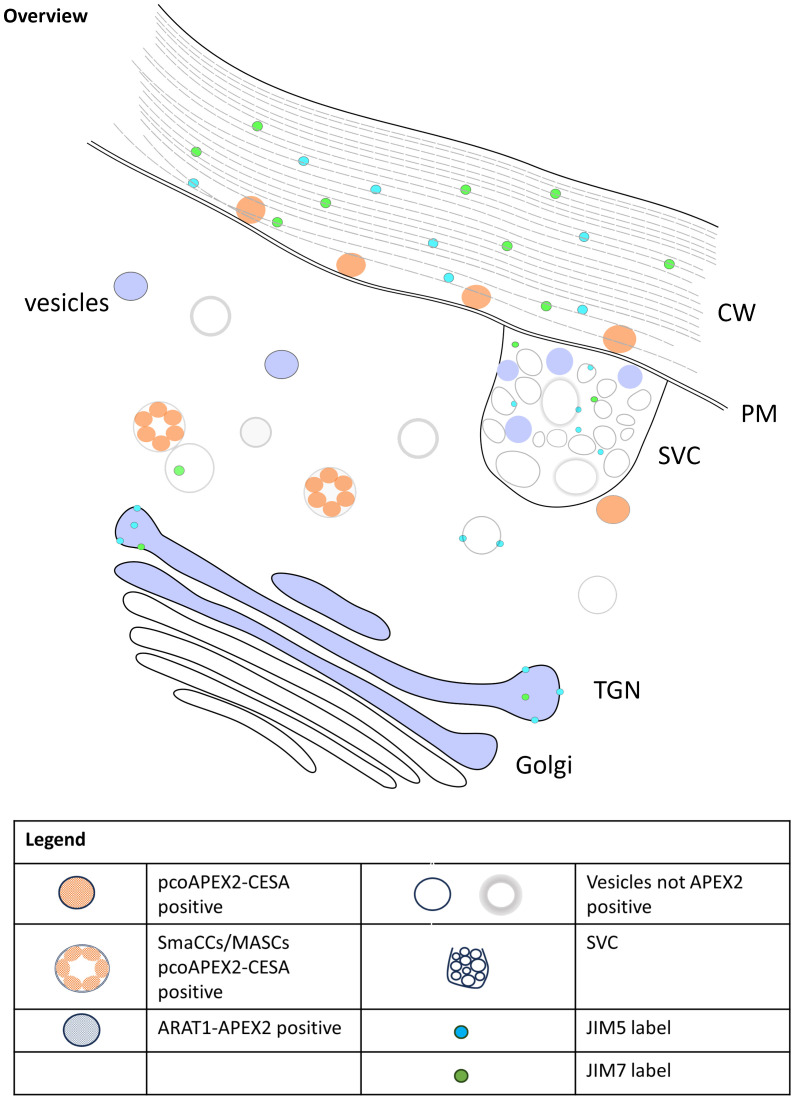
Model of trafficking of CESA6, *Na*ARADL1 and polysaccharides in *N. benthamiana*.

Our results, summarised in **Figure 4**, revealed distinct localization patterns for *At*CESA6 and *Na*ARADL1 within plant cells. APEX2-*At*CESA6 was predominantly localized to the plasma membrane, consistent with its role in cellulose synthesis. Interestingly, we also observed APEX2-*At*CESA6 densities in vesicles within the cytoplasm, suggestive of trafficking intermediates possibly corresponding to SmaCCs (Gutierrez et al., 2009) and/or MASCs (Crowell et al., 2009). This class of vesicles has been identified using fluorescence microscopy and is involved in the cycling of CSCs by both exo- and endo-cytosis (Zhu and McFarlane, 2022). We did not detect APEX2-*At*CESA6 densities at the GA, but this may be due to low abundance in the observed GA that did not include the APEX2-*At*CESA6 densities. In comparison, *Na*ARADL1-APEX2 exhibited localisation in the *trans*-Golgi cisternae, the TGN and cytoplasmic vesicles, some of which resemble SVCs, which is consistent with previous studies (Harholt et al., 2006; Lampugnani et al., 2016). These findings suggest distinct trafficking routes for cellulose synthases and arabinan transferases, highlighting the complexity of PCW biosynthesis and organization.

In conjunction with protein localization, we investigated the distribution of pectic polysaccharides using JIM5 and JIM7 antibodies. Both pectic antibodies were localised to the GA, specifically in this study the *trans*-Golgi cisternae, but previous studies have observed JIM5 labelling in the *cis*- and *medial*-Golgi cisternae as well (Zhang and Staehelin, 1992; Sinclair et al., 2018). Interestingly, JIM7 labelling exhibited a closer association with APEX2-*At*CESA6 densities at the plasma membrane compared to JIM5, suggesting potential co-trafficking of cellulose synthases and pectic polysaccharides. Furthermore, colocalization of pectic antibodies with *Na*ARADL1-APEX2 densities in secretory vesicle clusters (SVCs) suggests coordinated trafficking of arabinan transferases and pectic polysaccharides. Another possibility is that these vesicles may also serve as hubs for the recycling of ARADL1 proteins, facilitating the retrieval of ARADL1 proteins back to the Golgi apparatus or other cellular compartments involved in pectin metabolism. These observations align with previous studies demonstrating the co-trafficking of multiple polysaccharides in plant cells. For instance, Staehelin and colleagues showed that pectins and xyloglucans can be trafficked together (Lynch and Staehelin, 1992; Zhang and Staehelin, 1992). Additionally, as observed by Leucci et al. (2007), the distinct pathways followed by polysaccharides and secretory proteins further emphasize the intricate nature of PCW biosynthesis and secretion. While our study focused on protein and polysaccharide localization, previous research has identified key proteins like Syntaxin of Plants 61 (SYP61) involved in PCW trafficking (Drakakaki et al., 2012). The coordinated action of such proteins likely plays a crucial role in ensuring efficient PCW component delivery. Overall, our findings deepen our understanding of PCW dynamics and highlight the need for further research into the molecular interactions underlying PCW biosynthesis and secretion.

The application of this methodology can provide results that throw light on polysaccharide sorting in the trafficking pathways and can lead to the fine tuning of our understanding of polysaccharide and synthase pathways as they develop the PCW.

Our methodology offers several advantages for studying PCW biosynthesis and trafficking at the nanoscale. By genetically encoding protein tagging with APEX2 and combining it with polysaccharide localisation we achieved high-resolution visualization of PCW components. This approach provides powerful insights into the dynamics of PCW biosynthesis and trafficking, facilitating the elucidation of complex cellular processes. Additionally, APEX2 tagging circumvents the need for and complications arising from the use of additional antibodies, streamlining sample preparation and reducing the potential for artifacts associated with labelling of multiple antibodies on a section. For example, GFP is a commonly used translation tag, but labelling both GFP and multiple polysaccharides on a single sample would be challenging. APEX2 facilitates the simultaneous visualisation of multiple proteins and polysaccharides and enables comprehensive analyses of PCW dynamics at the TEM level, shedding light on intricate trafficking pathways and interactions between different components.

While there are many advantages to the use of APEX2 in plants, it is important to acknowledge the limitations of this methodology. The reliance on chemical fixation for APEX2 labelling may introduce artifacts, necessitating careful optimisation of fixation protocols to minimize potential distortions. Furthermore, the processing required to obtain electron densities using DAB staining may affect sample integrity and introduce variability in labelling efficiency. Future studies would benefit from the development of stable transformant lines, particularly in model plant species like Arabidopsis, to enable more consistent and reproducible analyses. Additionally, improved fixation techniques and complementary imaging modalities could enhance the accuracy and reliability of our observations, providing a more comprehensive understanding of PCW dynamics.

This study demonstrates the feasibility of employing a multi-pronged approach that combines protein tagging with APEX2 and polysaccharide localization to investigate PCW trafficking pathways at the nanoscale. Elucidation of the spatial organisation of PCW components within plant cells would advance our understanding of PCW biosynthesis and secretion processes. Moving forward, further refinement of this methodology and integration with complementary techniques will continue to enhance our ability to unravel the intricacies of PCW dynamics, paving the way for future discoveries in plant cell biology.

## Data Availability

The raw data supporting the conclusions of this article will be made available by the authors, without undue reservation.
