# Supplementary material for: Colocalising proteins and polysaccharides in plants for cell wall and trafficking studies
Source: Front Plant Sci. 2024 Sep 11;15:1440885. doi: 10.3389/fpls.2024.1440885 (PMC11425716; doi:10.3389/fpls.2024.1440885)
Supplement: Supplementary file 1 [file Table1.docx]

## Supplementary material 1 – construct details

### Sequence source

There are two versions of APEX, the original tag for electron microscopy, and APEX2 (Lam et al., 2015). APEX2 is the directed evolution sequence derived from the original Soybean sequence. This study aimed to use APEX2 in a dicotyledonous plant. The decision was made to codon optimise the sequence and we named this sequence pcoAPEX2.

To generate constructs containing pcoAPEX2 a double stranded DNA fragment was synthesised/ordered with the following sequence:

ACGCTCGAGGAATTCGGTACCATTATGGGAGCTGCTGCTGCTGCAGCTATGGGAAAGTCTTACCCTACTGTGTCTGCTGATTACCAGGATGCTGTTGAGAAGGCTAAGAAGAAGCTCAGGGGATTCATTGCTGAGAAGAGATGCGCTCCTCTCATGCTCAGACTCGCTTTTCATTCTGCTGGAACCTTCGATAAGGGAACTAAGACCGGTGGACCTTTCGGAACTATTAAGCACCCTGCTGAGCTTGCTCACTCTGCTAACAACGGACTCGATATCGCTGTTAGACTTCTCGAGCCTCTCAAGGCTGAGTTCCCTATCCTTTCTTACGCTGATTTCTACCAGCTCGCTGGTGTTGTTGCTGTTGAGGTTACAGGTGGACCTAAGGTGCCATTCCATCCTGGAAGAGAAGATAAGCCTGAACCTCCTCCAGAGGGTAGACTTCCTGATCCTACTAAGGGATCTGATCACCTCAGGGATGTGTTCGGAAAGGCTATGGGACTCACCGATCAGGATATCGTTGCTCTTTCTGGTGGACACACTATCGGAGCTGCTCACAAAGAGAGATCTGGATTTGAGGGACCTTGGACCTCTAACCCTCTCATCTTCGATAACTCTTACTTCACCGAGCTTCTCTCAGGTGAGAAAGAGGGACTTCTTCAGCTCCCTTCAGATAAGGCTCTCCTCTCTGATCCTGTGTTCAGACCTCTCGTGGATAAGTACGCTGCTGATGAGGATGCTTTCTTCGCAGATTACGCTGAGGCTCACCAGAAGCTTTCTGAGCTTGGATTTGCTGATGCTGGTGCAGCTGCTGCTGGTGCTCCTTAAGGATCCTCTAGAGTCCTGCTTTAAT

The resulting gene block included alanine linkers on the N and C terminus to facilitate cloning of fusions with genes of interest. The gene block was cloned into the *Kpn*I and *Bam*HI sites of the binary vector pFUERTE containing the CaMV35S promoter and the 3′ OCS terminator sequence (Lampugnani et al., 2016), using New England Biolabs HiFi DNA assembly reagents and the protocol specified by the manufacturer. This generated the construct 35S:pcoAPEX2. Details of generated constructs are available in Table S1.1 and the primers used are listed in Table S1.2.

**Table S1.1: Constructs generated in this study.**

| **Construct** | **Backbone** | **Bacteria** | **Resistance** |
| --- | --- | --- | --- |
| 35S:pcoAPEX2(empty) | pFUERTE | DH5A | KAN50 |
| 35S: pcoAPEX2-AtCESA6 | pFUERTE | DH5A | KAN50 |
| 35S:NaARAT1-pcoAPEX2 | pFUERTE | DH5A | KAN50 |

**Table S1.2: Primers utilised in this study.**

| **Primer Name** | **Primer Sequence** |
| --- | --- |
| CESA6 (pFUERTE) Rev | TAAAGCAGGACTCTAGAGgatccTCACAAGCAGTCTAAACCACAGATC |
| (AlaL) CESA6 Fwd | GCAGCTGCTGCTGGcgcc ATGAACACCGGTGGTCGGTT |
|  |  |
| (pFUERTE) NaARAT1 Fwd | GCTCGAGGAATTCGGTACc ATGGTTGAGAGAAATGCACATTCA |
| NaARAT1 (linker) Rev | TGCAGCAGCAGCgGCgCCCCAAAAATTTTTGGGCAACAG |
|  |  |
